# Supplementary material for: Facile Microwave Assisted Synthesis of Silver Nanostars for Ultrasensitive Detection of Biological Analytes by SERS
Source: Int J Mol Sci. 2022 Aug 8;23(15):8830. doi: 10.3390/ijms23158830 (PMC9369225; doi:10.3390/ijms23158830)
Supplement: Supplementary file 1 [file ijms-23-08830-s001.zip › ijms-1814775-supplementary.pdf]

Supplementary Materials

Facile Microwave Assisted Synthesis of Silver Nanostars for Ultrasensitive Detection of Biological Analytes by SERS

Radu Nicolae Revnic [1], Gabriela Fabiola Știufiuc [2,3,*], Valentin Toma [3], Anca Onaciu [3,4], Alin Moldovan [3], Adrian Bogdan Țigu [5], Eva Fischer-Fodor [6], Romulus Tetean [2], Emil Burzo [2] and Rareș Ionuț Știufiuc [3,4,*]

[1] Department of Family Medicine, "Iuliu Hatieganu" University of Medicine and Pharmacy, 2-4 Clinicilor Street, 400006 Cluj-Napoca, Romania

[2] Faculty of Physics, "Babes-Bolyai" University, 1 Kogalniceanu Street, 400084 Cluj-Napoca, Romania

[3] Department of BioNanoPhysics, MedFuture Research Center for Advanced Medicine, "Iuliu Hatieganu" University of Medicine and Pharmacy, 4-6 Pasteur Street, 400337 Cluj-Napoca, Romania

[4] Department of Pharmaceutical Physics & Biophysics, Faculty of Pharmacy, "Iuliu Hatieganu" University of Medicine and Pharmacy, 6 Pasteur Street, 400349 Cluj-Napoca, Romania

[5] Department of Translational Medicine, MedFuture Research Center for Advanced Medicine, "Iuliu Hatieganu" University of Medicine and Pharmacy, 4-6 Pasteur Street, 400337 Cluj-Napoca, Romania

[6] Oncology Institute "Prof. Dr. Ion Chiricuta", 400015 Cluj-Napoca, Romania

* Correspondence: gabriela.stiufiuc@ubbcluj.ro (G.F.Ș.); rares.stiufiuc@umfcluj.ro (R.I.Ș.); Tel.: +40-726-340-278 (R.I.Ș.)

S1. EDS Analysis

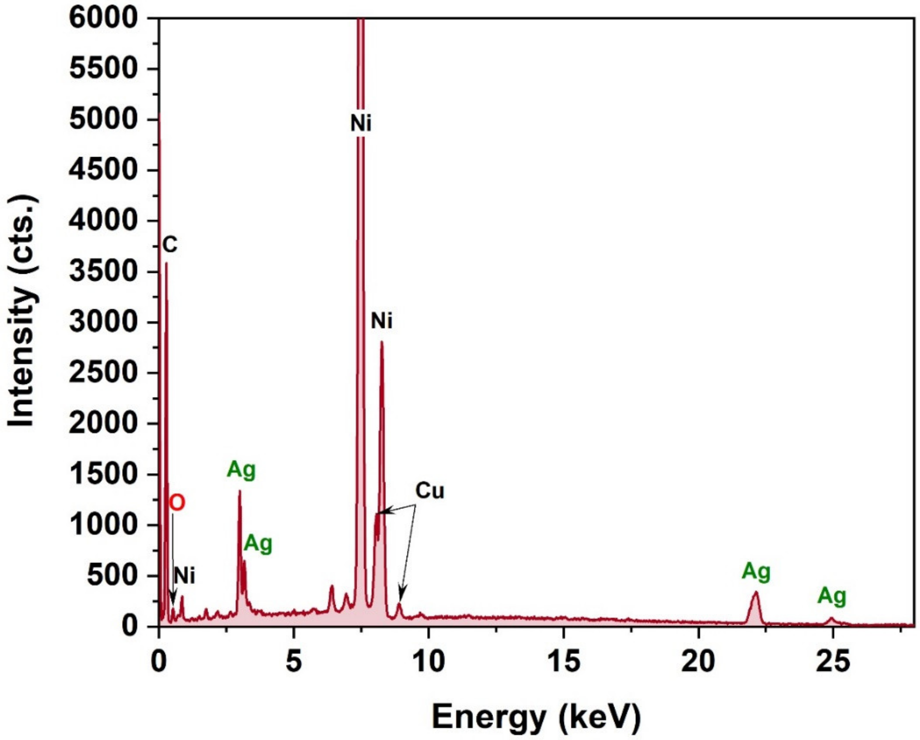

Figure S1. EDS analysis of purified silver colloids.

S2. Calculation of the EF

Rhodamine 6G (R6G) molecules have been employed as standard analytes for EF calculation. As such, we have recorded Raman and SERS spectra of R6G solution (1mM) under the same experimental conditions (Figure S1). The calculations have been performed according to the procedure developed by Gupta and Weimar [1], using the following equation:

$$EF = \frac{M_{Raman} \times S_{Surf} \times I_{Surf}}{M_{Surf} \times S_{Raman} \times I_{Raman}}$$

where M_{Surf} and M_{Raman} are the numbers of molecules, $S_{Surface}$ and S_{Raman} are the geometrical areas of the molecular films and I_{Surf} and I_{Raman} are the SERS/Raman intensities of the most intense vibrational band that has been used for the calculation of EF (1510 cm^{-1}). Both measurements were performed using a 50× objective and an excitation laser of 785 nm. In the case of Raman measurements, a 100% laser power was used, the acquisition time was 10 s and the number of spectral acquisitions was 4. In the case of SERS measurement, the only modification that has been made was the laser power which was set to 0.1%. The laser intensity, measured on sample surface, was 113 mW (100 % laser power) respectively 0.22 mW (0.1% laser power). The intensities of all vibrational bands included in this study were plotted in kcounts/(mW × s) units. In the case of Raman/SERS measurements employed for EF calculation 1mM aqueous solutions of R6G have been used. The diameter of the circular spot was ~2 mm in both cases. The Raman/SERS values of 1510 cm^{-1} band intensities were 43.3 respectively 0.0054 kcounts/(mW × s).

Using these data, the EF has a value of $\sim 8 \times 10^3$.

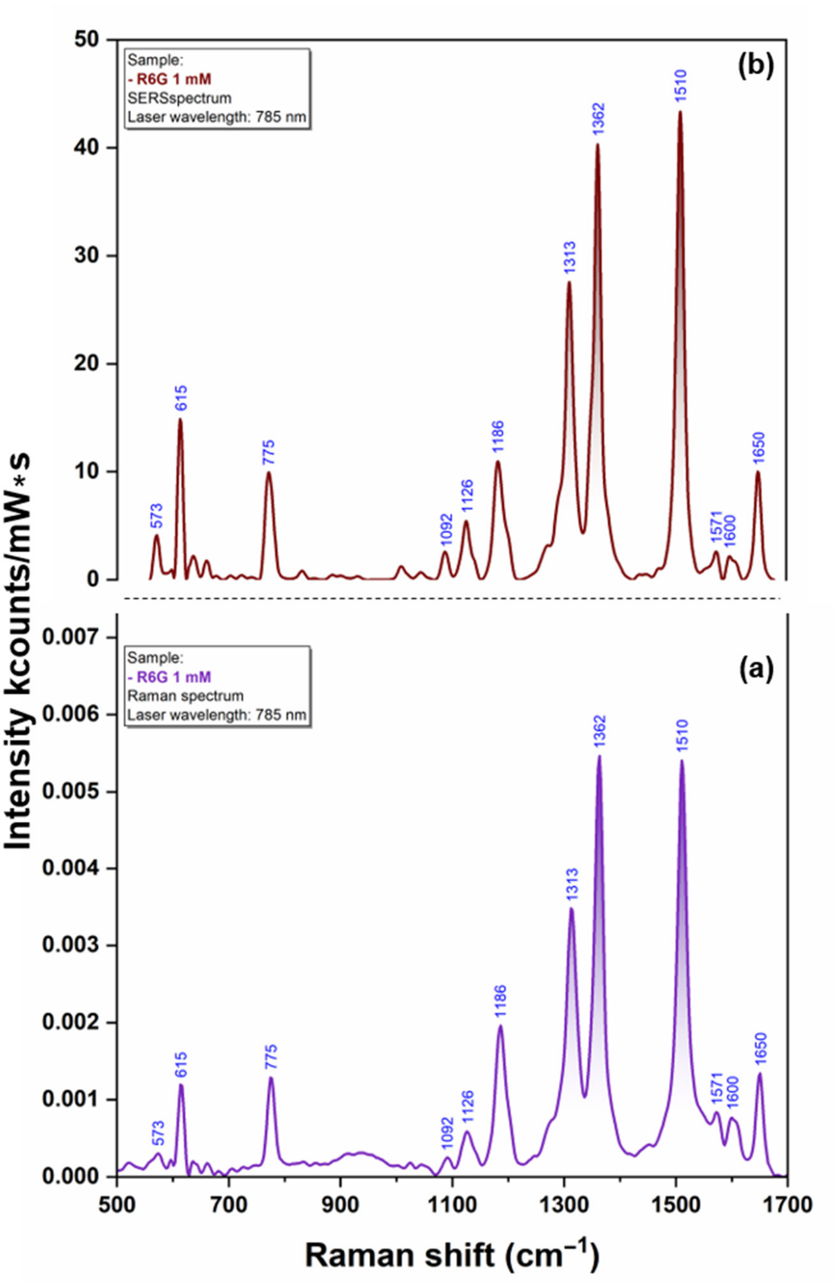

Figure S2. Raman (**a**) and SERS (**b**) spectra of R6G solutions (10^{-3} M) employed for EF calculation. The spectra were recorded using a 785 nm excitation laser.

Table S1. Tentative assignments of major vibrational bands in methylene blue, doxorubicin, atenolol, metoprolol, DLD1 cell lysates, cysteine, and methionine samples.

**Methylene Blue**		
Wavenumber (cm^{-1})	Vibration mode	References
451	In plane ring deformation $(C-N-C)_{AMG}$; Skeletal deformation $(C-N-C)$	[2–4]
504	In plane ring deformation $(C-N-C)_{AMG}$; Skeletal deformation $(C-N-C)$	[2–4]
599	In plane ring deformation $(C-N-C)_{AMG}$	[2,3]

615	Out of plane bend $(C-H)_{Ring}$	[2]
677	Out of plane bend $(C-H)_{Ring}$	[2,4]
773	Str $(C-N)_{AMG}$; In plane ring deformation $(C-N-C)_{Ring}$	[2,3]
831	Out of plane bend $(C-H)_{Ring}$	[2]
886	In plane ring deformation $(C-C-C)_{Ring}$	[2]
953	Rock (CH_2); In plane bend (CH)	[2,3]
1041	In plane bend (CH); Str $(C-S)$	[2,3]
1152	In plane bend (CH)	[2]
1178	Rock (CH_3); In plane bend (CH); Str $(C-N)$	[2,3,5]
1219	Rock (CH_3); In plane bend (CH)	[2]
1302	In plane bend (CH); Str $(C-N)_{Ring}$	[2,3]
1324	Str $(C-N)_{Ring}$; Str $(C-N)_{AMG}$; In plane bend (CH)	[2]
1395	Str (C_9-N_{10}); Str (C_3-N_2)	[2,3]
1433	In plane ring deformation $(N-C-H)_{AMG}$	[2]
1503	Twist (CH_2); In plane bend (CH)	[2]
**Doxorubicin**		
347	In plane deformation $(C-C-O)$	[6]
448	In plane deformation $(C=O)$; skeletal deformation	[6–8]
506	Skeletal deformation	[8]
917	Skeletal deformation	[8]
990	Str $(C-C)$; Ring breath	[7,8]
1211	In plane bend: $(O-H)$, $(C-O), (C-H), (C-O-H)$; Ring stretch symmetry	[7–9]
1241	In plane bend $(O-H)$; Ring stretch symmetry	[8]
1408	Skeletal ring stretching	[6–8]
1437	Bend $(CC-O-CH)$; Ring stretch	[8]
1456	Stretching at quinine ring; skeletal ring stretching; $(C-O-H)$ vibrations; In plane bend $(CC-O)$	[8–10]
1573	Ring stretching; Str $(C=C)$	[6–9]
1639	Hydrogen-bonded Str $(C=O)_{Ring}$; Hydrogen-bonded to OH group	[6,8,9]
**Atenolol**		
640	In plane bend $(C-C-C)_{Ring}$; Twist (N_5H_2); In plane ring deformation	[11,12]
722	Wag $(C-C-C)_{Ring}$; Rock (N_5H_2)	[11]
824	Rock (N_5H_2); In plane bend $(C-C-C)_{Ring}$; Out of plane bend $(C-H)_{Ring}$	[11,12]
859	Str (C_7C_9); Ring breathing	[11,12]
893	Str (C_7C_9); Ring breathing	[11]
950	Rock $(C_{18}H_{38})$; Wag $(C_{19}O_3N_5)$; In plane bend (CH_3)	[11,12]
1015	In plane bend $(CH)_{Ring}$; In plane ring deformation	[12]
1045	Rock (N_5H_2)	[11]
1160	In plane bend $(CH)_{Ring}$; Twist $(C_{18}H_2)$; In plane bend asymmetry (CH_2)	[11,12]
1230	Str (O_1C_{12}); In plane bend $(C-C-C)_{Ring}$	[11]

1240	In plane bend $(O_{10}H_2)$, $(C_{11}H_2)$, $(N_{12}H_2)$	[12]
1298	In plane bend $(C_{19}N_5H_{40})$; Wag $(C_{18}H_{12})$; In plane bend (CH_2)	[11,12]
1401	In plane bend: (CH_3), $(C_{11}H_2)$, (C_8H_2)	[12]
1453	In plane bend: $(C_{11}H_3)$, (C_9H_2), (CH_3)	[11,12]
1610	Str $(C-C)_{Ring}$; In plane bend $(C-H)_{Ring}$, (NH_2)	[11]
	**Metoprolol**	
639	In plane bend $(C-C-C)_{Ring}$; In plane ring deformation	[11,12]
662	In plane ring deformation	[12]
722	In plane bend: (NH), (CH), (CH_2), (CH_3)	[12]
754	In plane bend: (N_4H_{24}), $(C-C)_{Ring}$	[11]
827	Rock (C_5H_2), $(O_3H_{18}C_{19})$	[11]
852	Ring breathing; In plane bend (N_4H_{24})	[11,12]
920	In plane bend (CH_2), (CH_3)	[12]
949	Rock $(C_{11}H_2)$; In plane bend $(C_{10}H_3)$	[11]
1000	Str (C_8HC_9H); In plane bend: (CH_2), (CH_3)	[12]
1044	Str (O_1C_8)	[11]
1135	Rock: (CH_2), (CH_3), (NH_2)	[12]
1180	In plane bend: $(C_{13}H_{34})$, (O_2H_{35}); Rock: (CH_2), (CH_3)	[11,12]
1206	In plane bend: (O_2H_{35}), $(C_{13}H_{34})$	[11]
1240	Rock (CH_2)	[12]
1300	In plane bend: (C_5H_{20}), (OH), (NH), (CH_2); Wag (C_6H_2)	[11,12]
1341	In plane bend (OH); Twist (NH_2); Wag (CH_2)	[12]
1447	In plane bend: (C_9H_3), $(C_{10}H_3)$	[11]
1584	Str $(C-C)_{Ring}$; In plane bend $(C-H)_{Ring}$	[11,12]
1610	Str $(C-C)_{Ring}$; In plane bend $(C-H)_{Ring}$	[12]
	**DLD-1 cells lysate**	
627	Proteins, phenylalanine	[13]
656	Proteins, tyrosine	[14]
725	Bend $(N-H)$; Adenine; DNA	[15,16]
910	RNA	[15]
960	Proteins, tryptophan, valine	[14]
1094	Proteins: Str $(C-H)$	[15]
1278	Protein amide III	[14]
1328	Twist (CH_2); DNA; RNA	[16]
1371	Lipids: Symmetry bend(CH_3)	[17]
1396	Proteins: Twist (CH_2), (CH_3)	[18]
1453	(CH_2) bending mode in malignant tissues, bending modes of methyl groups (vibrational modes of collagen)	[16,19]
1541	(NH) and (NH_2) in cytosine, cytidine	[19]
1586	Scissor (CN_2) and rock (NH_2) in mitochondria and phosphorylated proteins	[19,20]

1695	In plane bend $(C = O)$ in nucleic acids	[13]
**Cysteine**		
665	Scissor $(C_\gamma O_2^-)$; Str $(C_\beta - S)$	[21]
810	Bend $(C_\gamma C_\alpha C_\beta)$; Str $(C_\alpha - S)$; Str $(C_\alpha - C_\beta)$	[21]
830	Wag $(C_\beta H_2)$; Str $(C_\alpha - N)$	[21]
908	Str $(C_\alpha - C_\gamma)$; Rock (NH_3^+); Scissor $(C_\gamma O_2^-)$	[21]
1001	In plane bend (CCN); Bend (NCH)	[22]
1054	Twist $(C_\beta H_2)$; Str $(C_\alpha - N)$	[21]
1132	Str $(C_\alpha - C_\beta)$; Rock (NH_3^+); Str $(C_\alpha - N)$	[21]
1234	Wag $(C_\beta H_2)$; Bend $(C_\alpha - H)$	[21]
1286	Bend $(C_\alpha - H)$; Wag $(C_\beta H_2)$	[21]
1349	Symmetric Str $(C_\gamma O_2^-)$; Bend $(C_\alpha - H)$	[21]
1415	Symmetric Str $(C_\gamma O_2^-)$	[21]
1519	In plane bend (COO^-)	[22]
1596	Asymmetric Str $(C_\gamma O_2^-)$; Bend (NH_3^+)	[21]
**Methionine**		
560	Rock (CO_2^-)	[23]
682	Str $(C_4 - S)$	[24]
753	Rock (CO_2^-)	[23]
829	Asymmetric Str $(C - S - C)$	[25]
856	Str $(C_2 - C_3)$	[24]
950	Str $(C_1 - C_2)$	[24]
1002	Rock (CH_2)	[23]
1049	Str $(C_2 - N)$	[24]
1141	Asymmetric Str $(C_\alpha CN)$	[25]
1238	In plane bend $(CC_\alpha H)$	[25]
1283	Twist (CH_2)	[23]
1318	Str $(C - NH_2)$	[25]
1382	Str (COO^-)	[25]
1444	In plane bend asymmetry (CH_3); In plane bend symmetry (CH_2)	[23]

Abbreviations: AMG -attached to methyl group; Str- stretching

References

1. Gupta, R.; Weimer, W.A. High enhancement factor gold films for surface enhanced Raman spectroscopy. *Chem. Phys. Lett.* **2003**, *374*, 302–306. https://doi.org/10.1016/S0009-2614(03)00737-1.
2. Dutta Roy, S.; Ghosh, M.; Chowdhury, J. Adsorptive parameters and influence of hot geometries on the SER(R) S spectra of methylene blue molecules adsorbed on gold nanocolloidal particles. *J. Raman Spectrosc.* **2015**, *46*, 451–461. https://doi.org/10.1002/jrs.4675.

3. Ikramova, S.B.; Utegulov, Z.N.; Dikhanbayev, K.K.; Gaipov, A.E.; Nemkayeva, R.R.; Yakunin, V.G.; Savinov, V.P.; Timoshenko, V.Y. Surface-Enhanced Raman Scattering from Dye Molecules in Silicon Nanowire Structures Decorated by Gold Nanoparticles. *Int. J. Mol. Sci.* **2022**, *23*, 2590. https://doi.org/10.3390/ijms23052590.
4. Ruan, C.; Wang, W.; Gu, B. Single-molecule detection of thionine on aggregated gold nanoparticles by surface enhanced Raman scattering. *J. Raman Spectrosc.* **2007**, *38*, 568–573. https://doi.org/10.1002/jrs.1691.
5. Harpster, M.H.; Zhang, H.; Sankara-Warrier, A.K.; Ray, B.H.; Ward, T.R.; Kollmar, J.P.; Carron, K.T.; Mecham, J.O.; Corcoran, R.C.; Wilson, W.C.; et al. SERS detection of indirect viral DNA capture using colloidal gold and methylene blue as a Raman label. *Biosens. Bioelectron.* **2009**, *25*, 674–681. https://doi.org/10.1016/j.bios.2009.05.020.
6. Eliasson, C.; Lorén, A.; Murty, K.V.G.K.; Josefson, M.; Käll, M.; Abrahamsson, J.; Abrahamsson, K. Multivariate evaluation of doxorubicin surface-enhanced Raman spectra. *Spectrochim. Acta - Part A Mol. Biomol. Spectrosc.* **2001**, *57*, 1907–1915. https://doi.org/10.1016/S1386-1425(01)00453-X.
7. Chul, J.L.; Jae, S.K.; Mak, S.K.; Kwang, P.L.; Mu, S.L. The study of doxorubicin and its complex with DNA by SERS and UV-resonance Raman spectroscopy. *Bull. Korean Chem. Soc.* **2004**, *25*, 1211–1216. https://doi.org/10.5012/bkcs.2004.25.8.1211.
8. Gautier, J.; Munnier, E.; Douziech-Eyrolles, L.; Paillard, A.; Dubois, P.; Chourpa, I. SERS spectroscopic approach to study doxorubicin complexes with Fe2+ ions and drug release from SPION-based nanocarriers. *Analyst* **2013**, *138*, 7354. https://doi.org/10.1039/c3an00787a.
9. Beljebbar, A.; Sockalingum, G.D.; Angiboust, J.F.; Manfait, M. Comparative FT SERS, resonance Raman and SERRS studies of doxorubicin and its complex with DNA. *Spectrochim. Acta Part A Mol. Spectrosc.* **1995**, *51*, 2083–2090. https://doi.org/10.1016/0584-8539(95)01515-7.
10. Minati, L.; Maniglio, D.; Benetti, F.; Chiappini, A.; Speranza, G. Multimodal Gold Nanostars as SERS Tags for Optically-Driven Doxorubicin Release Study in Cancer Cells. *Materials (Basel).* **2021**, *14*, 7272. https://doi.org/10.3390/ma14237272.
11. Farcas, A.; Iacovita, C.; Vinteler, E.; Chis, V.; Stiufiuc, R.; Lucaciu, M. The Influence of Molecular Structure Modifications on Vibrational Properties of Some Beta Blockers: A Combined Raman and DFT Study. *J Spectrosc* **2016**, *2016*, 1–9. https://doi.org/10.1155/2016/3137140.
12. Cozar, I.B.; Szabó, L.; Leopold, N.; Chiş, V.; David, L. Raman, sers and dft study of atenolol and metoprolol cardiovascular drugs. *Rom. Reports Phys.* **2010**, *55*, 772–781.
13. Guerrini, L.; Garcia-Rico, E.; O'Loghlen, A.; Giannini, V.; Alvarez-Puebla, R.A. Surface-Enhanced Raman Scattering (SERS) Spectroscopy for Sensing and Characterization of Exosomes in Cancer Diagnosis. *Cancers (Basel).* **2021**, *13*, 2179. https://doi.org/10.3390/cancers13092179.
14. Bruzas, I.; Lum, W.; Gorunmez, Z.; Sagle, L. Advances in surface-enhanced Raman spectroscopy (SERS) substrates for lipid and protein characterization: sensing and beyond. *Analyst* **2018**, *143*, 3990–4008. https://doi.org/10.1039/C8AN00606G.
15. Liu, M.; Liu, X.; Huang, Z.; Tang, X.; Lin, X.; Xu, Y.; Chen, G.; Kwok, H.F.; Lin, Y.; Feng, S. Rapid discrimination of colon cancer cells with single base mutation in KRAS gene segment using laser tweezers Raman spectroscopy. *J. Biophotonics* **2019**, *12*. https://doi.org/10.1002/jbio.201800332.
16. Cao, Z.; Pan, X.; Yu, H.; Hua, S.; Wang, D.; Chen, D.Z.; Zhou, M.; Wu, J. A Deep Learning Approach for Detecting Colorectal Cancer via Raman Spectra. *BME Front.* **2022**, *2022*, 1–10. https://doi.org/10.34133/2022/9872028.

17. Liu, W.; Sun, Z.; Chen, J.; Jing, C. Raman Spectroscopy in Colorectal Cancer Diagnostics: Comparison of PCA-LDA and PLS-DA Models. *J. Spectrosc.* **2016**, *2016*, 1–6. https://doi.org/10.1155/2016/1603609.
18. Kneipp, J.; Kneipp, H.; Wittig, B.; Kneipp, K. Novel optical nanosensors for probing and imaging live cells. *Nanomedicine Nanotechnology, Biol. Med.* **2010**, *6*, 214–226. https://doi.org/10.1016/j.nano.2009.07.009.
19. Brozek-Pluska; Musial; Kordek; Abramczyk Analysis of Human Colon by Raman Spectroscopy and Imaging-Elucidation of Biochemical Changes in Carcinogenesis. *Int. J. Mol. Sci.* **2019**, *20*, 3398. https://doi.org/10.3390/ijms20143398.
20. Beton, K.; Wysocki, P.; Brozek-Pluska, B. Mevastatin in colon cancer by spectroscopic and microscopic methods – Raman imaging and AFM studies. *Spectrochim. Acta Part A Mol. Biomol. Spectrosc.* **2022**, *270*, 120726. https://doi.org/10.1016/j.saa.2021.120726.
21. Yao, G.; Huang, Q. DFT and SERS Study of <scp>l</scp> -Cysteine Adsorption on the Surface of Gold Nanoparticles. *J. Phys. Chem. C* **2018**, *122*, 15241–15251. https://doi.org/10.1021/acs.jpcc.8b00949.
22. Chang, C.; Chen, Y.; Huang, Y.; Lai, C.-H.; Jeng, U.-S.; Lai, Y.-H. Nanostructured silver dendrites for photon-induced Cysteine dimerization. *Sci. Rep.* **2019**, *9*, 20174. https://doi.org/10.1038/s41598-019-56517-5.
23. Melo, W.D.C.; Freire, P.T.C.; Filho, J.M.; Melo, F.E.A.; Lima, J.A.; Paraguassu, W. Raman spectroscopy of d-methionine under high pressure. *Vib. Spectrosc.* **2014**, *72*, 57–61. https://doi.org/10.1016/j.vibspec.2014.02.012.
24. Graff, M.; Bukowska, J. Surface-enhanced Raman scattering (SERS) spectroscopy of enantiomeric and racemic methionine on a silver electrode-evidence for chiral discrimination in interactions between adsorbed molecules. *Chem. Phys. Lett.* **2011**, *509*, 58–61. https://doi.org/10.1016/j.cplett.2011.04.089.
25. Podstawka, E.; Ozaki, Y.; Proniewicz, L.M. Part II: Surface-Enhanced Raman Spectroscopy Investigation of Methionine Containing Heterodipeptides Adsorbed on Colloidal Silver. *Appl. Spectrosc.* **2004**, *58*, 581–590. https://doi.org/10.1366/000370204774103417.
